# A Learning Framework for Atomic-Level Polymer Structure Generation

**DOI:** 10.1021/acs.chemmater.5c01644

**Published:** 2025-09-11

**Authors:** Ayush Jain, Ashutosh Srivastava, Rampi Ramprasad

**Affiliations:** † School of Materials Science and Engineering, 1372 Georgia Institute of Technology, 771 Ferst Drive, Atlanta, Georgia 30332, United States; ‡ School of Computational Science and Engineering, Georgia Institute of Technology, 756 W Peachtree St NW, Atlanta, Georgia 30332, United States

## Abstract

Synthetic polymeric materials underpin fundamental technologies in the energy, electronics, consumer goods, and medical sectors, yet their development still suffers from prolonged design timelines. Although polymer informatics tools have supported speedup, polymer simulation protocols continue to face significant challenges in the on-demand generation of realistic 3D atomic structures that respect the conformational diversity of polymers. Generative algorithms for 3D structures of inorganic crystals, biopolymers, and small molecules exist, but have not addressed synthetic polymers because of challenges in representation and data set constraints. In this work, we introduce polyGen, a generative model designed specifically for 3D polymer structures that operates from minimal inputs such as the repeat unit chemistry alone. polyGen combines graph-based encodings with a latent diffusion transformer using positional biased attention for realistic conformation generation. Given the limited data set of 3,855 DFT-optimized polymer structures, we incorporate joint training with small molecule data to enhance generation quality. We also establish structure matching criteria to benchmark our approach on this novel problem. polyGen overcomes the limitations of traditional crystal structure prediction methods for polymers, successfully generating realistic and diverse linear and branched conformations, with promising performance even on challenging large repeat units. As an atomic-level proof-of-concept capturing intrinsic polymer flexibility, it marks a transformative capability in material structure generation.

## Graphical abstract

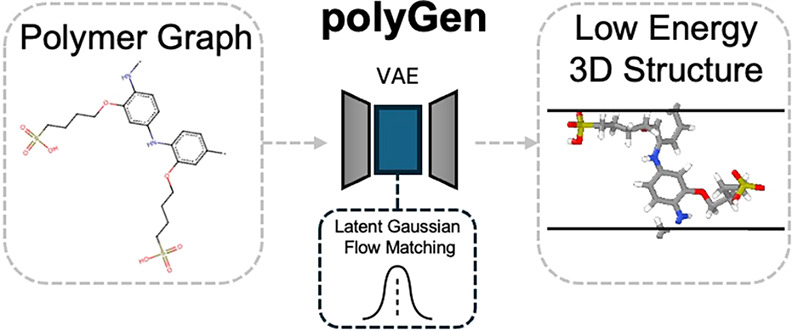

## Introduction

Polymeric materials play a central role in modern science and engineering, enabling technologies across sectors such as packaging, electronics, medicine, and energy.^1,2^ Their versatility arises from the vast structural diversity of organic building blocks,^3^ the ingenuity of synthetic chemistry, and the breadth of accessible processing techniques. By tuning parameters such as monomer composition, chain architecture, additives, and processing conditions, polymers can be engineered to span a wide range of mechanical, electrical, and transport properties—from rigid plastics, elastomers, dielectrics,^4^ and membranes.^5–7^ Despite the impact of polymers, the discovery and deployment of new materials remains a slow and resource-intensive process. This is due to the vastness of chemical and processing design spaces^8^ and the need to balance performance, cost, safety, and manufacturability. As a result, polymer innovation still relies heavily on experience, trial-and-error, chemical intuition, and serendipity. Novel methods of generating polymer designs guided by informatics approaches have emerged, such as virtual forward synthesis, evolutionary algorithms, and syntax-directed autoencoders that can produce a theoretically innumerable amount of polymer candidates.^1,9–11^ Physics-based computer simulations may accelerate the pace of polymer discovery but these methods also face barriers that have thus far prevented the widespread utilization of such approaches as detailed below.

A key challenge faced by the polymer simulation community is the creation of suitable initial atomic-level structures, especially for novel chemistries. Unlike crystalline and inorganic materials, polymers exhibit a complex combination of amorphous, semicrystalline, and crystalline domains, with conformational flexibility and structural disorder playing essential roles in their function.^12^ The present work serves as a concrete proof-of-concept demonstrating that ground state single-chain polymer conformations may be reliably and consistently generated in a time-efficient manner. Expensive Density Functional Theory (DFT) calculations were used to produce training data for a diverse set of single-chain chemistries.^12,13^ Going beyond ground state single chains using DFT is an expensive task. There is a growing need for predictive tools that can generate realistic 3D atomic structures of polymers from minimal inputs, such as chemical composition or connectivity (e.g., SMILES), as shown in Figure 1. Such tools would serve as a rapid and diverse structure generation start for downstream DFT and molecular simulation tasks, enabling property prediction and rational design faster in the discovery pipeline.

**Figure 1:**
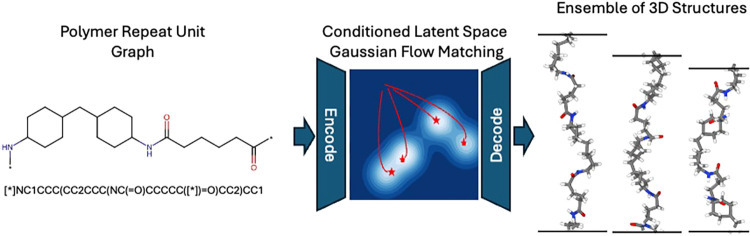
Theoretical overview of polyGen from the perspective of chemistry-conditioned energy minimization of the potential energy surface represented in the latent space. With this capability, an initial connectivity for a polymer repeat unit can be used to generate structures with a high probability of being at a potential energy minimum.

### Generative Materials Structure Prediction

Materials generation via diffusion models^14^ or flow matching^15^ offers promise. Typically, these models use data sets containing structures optimized by DFT^16^ or Classical MD simulations.^17^ With materials research, these methods have been used thus far for inorganic crystalline materials with a finite number of atoms within a unit cell parametrized by the lattice angles and lengths.^18–20^ Significant progress has also emerged in structure prediction for large biological polymers, particularly proteins, where models like AlphaFold^17,21^ and Boltz-1 ^22^ can now generate accurate three-dimensional conformations from amino acid sequences. Similarly, the generation of small molecule conformers^23–25^ has also been an open problem, especially in the context of protein docking.^26^

The synthetic polymers generation problem is a mix of all of these applications. Similar to crystals, synthetic polymers can be defined by a periodic unit. 3D structures of these repeat units are also defined within unit cells/bounding boxes.^12^ Traditional crystal structure prediction methods, while powerful for smaller systems, have not displayed abilities to capture the rich conformational landscape of polymers. The local features of synthetic polymers are more akin to molecules where the local (short-range) connectivity of their structures is well-defined. However, polymers occur as long chains composed of sequences of 10s-10,000s of monomeric repeat units, which necessitates an understanding of longer-range interactions for stochastic structure generation. This is similar to protein modeling approaches like AlphaFold3, which employs transformers to predict an ensemble of structures.^21^ However, unlike proteins, which have a consistent backbone (a sequence of repeating *N* – *C*_α_ – *C*, where *C*_α_ is the centrally located carbon in the amino acid residue) and a finite set of amino acids, synthetic polymers boast a limitless design space to draw their repeat units and backbones from.^1,2^ Additionally, proteins and molecules are aperiodic and nonperiodic, respectively. In combination with the aforementioned challenges, polymer structure data sets from MD or DFT have only recently been standardized and have not seen the scale required for generative modeling.^12^ Moreover, past work^27^ in general may overlook the need of representing chain level,^12^ amorphous,^5,28^ and network systems,^29^ in simulations using periodic boundary conditions. Strategies like the tokenization of polymer repeat units may work in contexts of linear chains, but inconsistent polymers such as thermosets and ladder polymers cannot always be discretized in this way. To have future capabilities in this direction, we need an all-atom framework that can be extended to irregular and complex structures.

The complexity of polymer design and representation, combined with limited available data, explains why generative models for polymers have remained largely unexplored until now. We introduce polyGen (Figure 2), the first latent diffusion model specifically designed to generate periodic and stochastic polymeric structures with an all-atom approach. We build upon latent diffusion frameworks for materials generation such as All-Atom DiT.^30^ Our problem and approach are modified to predict an ensemble of low-energy polymer conformations conditioned on a specified repeat unit with prescribed atomic connectivities rather than performing unconstrained de novo structure generation. Our method leverages a molecular encoding that captures atomic connectivity, which is used as a conditioning signal throughout the model architecture. We adopt a latent diffusion strategy over joint diffusion methods such as DiffCSP^18^ or molecular generation methods in Cartesian space^23^ due to the strong coupling between atomic positions and box dimensions imposed by polymer connectivity constraints. We augment our data set with smaller structures from DFT-optimized molecules, demonstrating improved polymer structure prediction capabilities owing to the shared weights of a data set invariant encoder and decoder, and a shared latent space. This work culminates in a learning framework that can generate ensembles of realistic polymer chain conformations. To benchmark the quality of these conformations, we introduce rigorous evaluation criteria on bonds, angles, and dihedrals—standards that, to our knowledge, have not yet been applied in the context of materials generation. This application represents a proof-of-concept to predict atomic-level synthetic polymer conformations while accounting for their intrinsic flexibility and the distribution of plausible structures.

**Figure 2:**
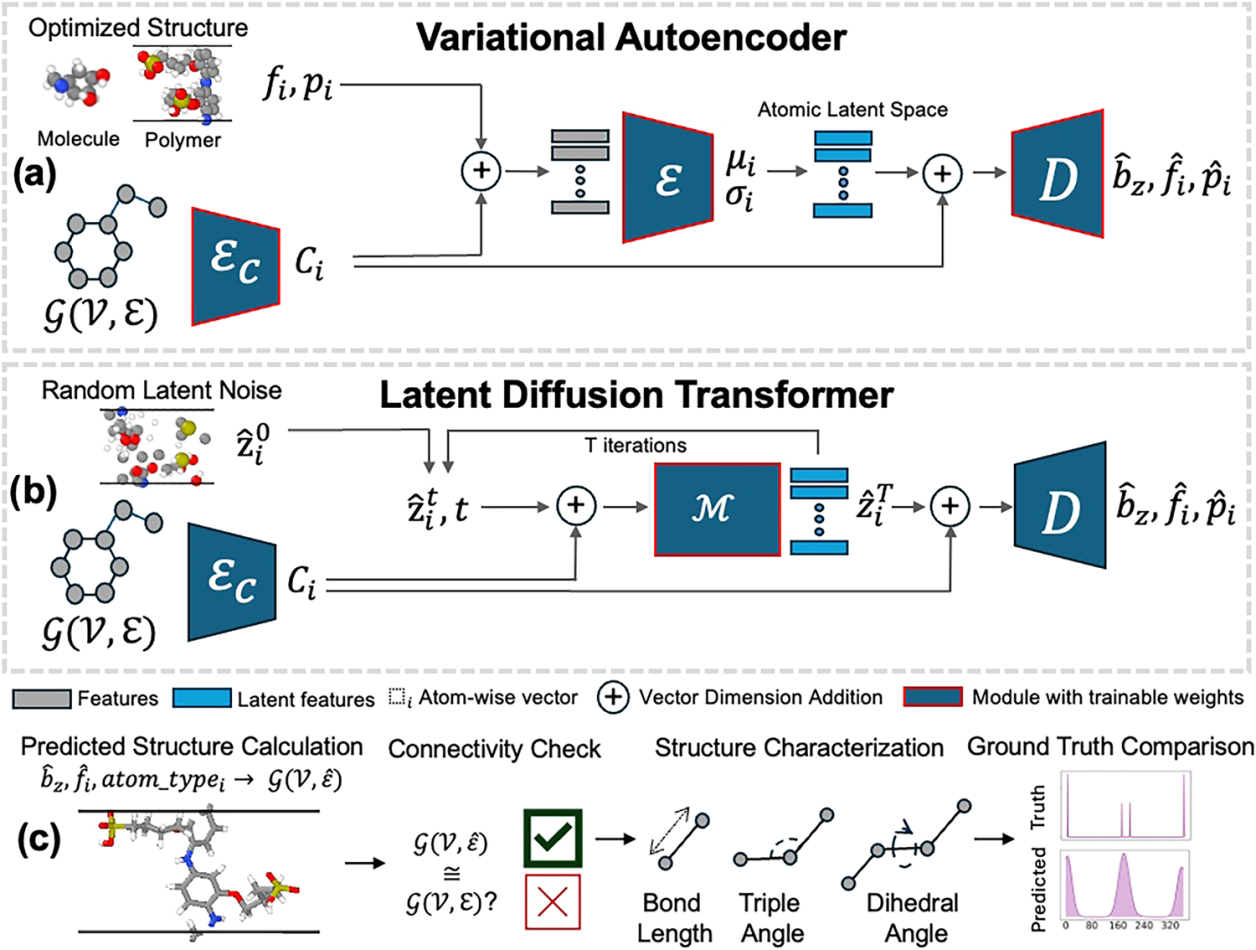
Training and generation process for polyGen. (a) First, an autoencoder with conditional encoder $E_{C}$ , encoder E , and decoder D is trained to learn an atom-wise latent space by reconstructing the system as a bounding box *b̂*_*z*_, fractional coordinates *f*_*i*_, and Cartesian coordinates *p*_*i*_. In this process, an atom-wise conditioning *C*_*i*_ is learned. (b) The diffusion model M is trained within this latent space, iteratively denoising a Gaussian random latent *ẑ*_*i*_^0^ into a new distribution *ẑ*_*i*_^*T*^ that is likely a valid polymer conformation. (c) During postgeneration filtering, a structure is calculated from the predicted positions, and can be used if connectivity and bonding are preserved. The structure is characterized to obtain a predicted bond, angle, and dihedral distributions.

## Methods

polyGen consists of three phases, a 0D conditioning on the molecular graph of the desired polymer repeat unit chemical structure, a variational autoencoder for structure (Figure 2a), and a latent diffusion module (Figure 2b). Overall, we choose an architecture that does not include any equivariance, following the sentiment of recent works^21,30^ where data scale and augmentation can be a path to learning rotational equivariance efficiently. Instead, we focus on an architecture that biases our predictions toward polymer structure, as this is the more challenging aspect of the learning problem.

### Polymer Structure Specification

Several previous works have learned structural and chemical representations of periodic materials, which include fractional coordinates, unit cell lengths, and lattice angles. For single polymer chain structures, we take a modified approach. Because of their complexity, training set structures are optimized using DFT with a periodic orthogonal box where the chain is continuous through the *z* axis. The width of the box is needed to encapsulate side chains/branched structures and are made large enough to isolate the single chain during optimization.

We note that the *x* and *y* axis are not entirely polymer structure dependent. Including these as prediction variables is an overparameterization, and does not contribute much information. Therefore, we fix the *x* and *y* of the box to 55 Å × 55 Å, and define the system by the *z* height of the box, *b*_*z*_. In theory, this quantity is a proxy for the density of the chain conformation and could provide insights into the contour length projection and free volume of the polymer chain. For example, a rigid chain with the same number of atoms in the unit cell will likely have a longer *b*_*z*_ due to lack of flexibility, whereas flexible chains can compress into denser arrangements with reduced *z* height. Atomic positions are provided as fractional coordinates within this orthogonal bounding box.

### Graph Conditioning

The generation of polymer structures begins with a minimal representation of atomic connectivity, typically provided in the form of a SMILES string. This string is converted into a molecular graph: periodic for polymers and nonperiodic for small molecules. To extract a universal atom-wise representation, denoted as *C*_*i*_, we apply a graph conditioning module that encodes the molecular connectivity. As illustrated in Figure 2a,b, *C*_*i*_ serves as contextual input to the encoder, decoder, and diffusion transformer modules. The graph conditioning module is implemented using a graph interaction network, similar to,^31^ which captures local chemical environments by modeling interactions between different atom and bond types. Importantly, the same network weights are used across all data sets and connectivity types, facilitating effective transfer learning and generalization across chemical spaces.

Given a graph G=(V,E) , each node i∈V is associated with an atom type and positional encodings, and each edge (i,j)∈E is associated with a bond type. The initial node and edge embeddings are computed as

1 $$h_{i}^{\left(0\right)}=\mathrm{Embed}\left(\mathrm{atom}\_{\mathrm{type}}_{i}\right),e_{\mathrm{ij}}^{\left(0\right)}=\mathrm{Embed}\left(\mathrm{bond}\_{\mathrm{type}}_{\mathrm{ij}}\right),h_{i}^{\left(0\right)}=\mathrm{MLP}\left([h_{i}^{\left(0\right)};r_{i}^{\mathrm{RW}};r_{i}^{\mathrm{Lap}}]\right)$$

For each layer *l* = 1, ..., *L*, we update the edges and the nodes as

2 $$\begin{array}{l} e_{\mathrm{ij}}^{\left(l\right)}=e_{\mathrm{ij}}^{\left(l-1\right)}W_{e}+f_{e}^{\left(l\right)}\left(\mathrm{LN}\left([e_{\mathrm{ij}}^{\left(l-1\right)};h_{i}^{\left(l-1\right)};h_{j}^{\left(l-1\right)}]\right)\right)\\ m_{i}^{\left(l\right)}=\sum_{j\in N\left(i\right)}e_{\mathrm{ij}}^{\left(l\right)},h_{i}^{\left(l\right)}=h_{i}^{\left(l-1\right)}W_{v}+f_{v}^{\left(l\right)}\left(\mathrm{LN}\left([m_{i}^{\left(l\right)};h_{i}^{\left(l-1\right)}]\right)\right) \end{array}$$

We aggregate node features to add global context for the whole molecule/polymer:

3 $$g=\sum_{i\in V}h_{i}^{\left(L\right)},C_{i}=f_{f}^{\left(l\right)}\left([g;h_{i}^{\left(L\right)}]\right)$$

where *f*_*e*_^(*l*)^, *f*_*v*_^(*l*)^, *f*_*f*_^(*l*)^ are MLPs, LN is layer normalization, **r**_*i*_^RW^ is a random walk positional encoding^32^ of size 16 and **r**_*i*_^Lap^ is a Laplacian positional encoding^33,34^ of size 2. We use *L* = 4 to capture local interactions of an atom 1 “hop” away from its furthest dihedral. The global pooling prior to embedding into token dimension is done so that atomic-level information is taken with global system information.

The encoder, decoder, and diffusion modules are all transformers, which typically require a positional encoding to identify token ordering and condition the self-attention weights. Similar to contemporary graph transformer methods, *C*_*i*_ communicates this by providing embeddings calculated from **r**_*i*_^RW^ and **r**_*i*_^Lap^.^35^

### Structural Variational AutoEncoder

The next step is to learn a way to combine all aspects of a polymer system, such as monomer chemistry, fractional coordinates, and the bounding box, into a joint space. This space should also allow the fusion of information from small molecules or other systems to enhance learning. We employ a VAE to create unified atom-wise latent representation that contains structural details for small molecules and polymers, $Z\in R^{d}$ where *d* is a latent dimension. The VAE is based on traditional graph VAEs, including an encoder E and a decoder D operating atom-wise.

The structure of the molecule/polymer is given through a concatenated vector of fractional coordinates *f*_*i*_ and Cartesian positions *p*_*i*_. For a polymer structure, both the *f*_*i*_ and scaled *p*_*i*_ according to the bounding box are provided. This forces the VAE to learn and reconstruct the relationship between the Cartesian positions, fractional coordinates, and the bounding box. For nonperiodic molecules, *p*_*i*_ is provided and *f*_*i*_ = ⌀, which allows periodic and nonperiodic materials to share the same latent space, similar to.^30^ For example, we set *f*_*i*_ = ⌀ for samples from the QM9 data set. The atom-wise latent space is then calculated along with the *C*_*i*_.

4 $$\mu_{i},\sigma_{i}=E\left(f_{i},p_{i},C_{i}\right)$$

5 $$Z_{i}∼N\left(\mu_{i},\sigma_{i}\right)$$

The decoder then produces a structure from Z

6 $${\mathrm{f̂}}_{i},{\mathrm{p̂}}_{i},{\mathrm{b̂}}_{z}=D\left(Z,C_{i}\right)$$

where *f̂*_*i*_ is the predicted fractional coordinates, *p̂*_*i*_ is the predicted Cartesian coordinates, and *b̂*_*z*_ is the *z* direction height of the bounding box. The loss on the decoder is used to optimize the model

7 $$L_{\mathrm{total}}=\left\langle w_{\mathrm{bbox}}\cdot L_{\mathrm{bbox}}\right\rangle +\left\langle w_{\mathrm{frac}\_\mathrm{coords}}\cdot L_{\mathrm{frac}\_\mathrm{coords}}\right\rangle +\left\langle w_{\mathrm{pos}}\cdot L_{\mathrm{pos}}\right\rangle +\left\langle w_{\mathrm{kl}}\cdot L_{\mathrm{kl}}\right\rangle +\left\langle w_{\mathrm{bond}}\cdot L_{\mathrm{bond}}\right\rangle +\left\langle w_{\mathrm{angle}}\cdot L_{\mathrm{angle}}\right\rangle +\left\langle w_{\mathrm{dihedral}}\cdot L_{\mathrm{dihedral}}\right\rangle$$

Where the reconstruction terms are defined as

$$\begin{array}{l} L_{\mathrm{bbox}}=\mathrm{MSE}({\mathrm{b̂}}_{z},b_{z}/\left(10\sqrt[{3}]{N}\right)),L_{\mathrm{frac}\_\mathrm{coords}}=\frac{1}{d}\sum_{i}\mathrm{MSE}\left({\mathrm{f̂}}_{i},f_{i}\right)\\ L_{\mathrm{pos}}=\frac{1}{d}\sum_{i}\mathrm{MSE}\left({\mathrm{p̂}}_{i}-\left\langle \mathrm{p̂}\right\rangle ,p_{i}-\left\langle p\right\rangle \right),L_{\mathrm{kl}}=\mathrm{KL}\left(q_{\phi }\left(z|x\right)∥p\left(z\right)\right) \end{array}$$

where $L_{\mathrm{bbox}}=0,L_{\mathrm{pos}=0}$ for periodic structures and $L_{\mathrm{frac}\_\mathrm{coords}}$ =0 for nonperiodic structures. The scaling factor $b_{z}/\left(10\sqrt[{3}]{N}\right)$ aligns with previous work,^18^ and forces polyGen to be invariant to the number of atoms in the system and use the conditioning atomic connectivity to estimate *b*_*z*_. The structural loss terms are

$$L_{\mathrm{bond}}=\mathrm{MSE}\left(d_{\mathrm{ij}},{\mathrm{d̂}}_{\mathrm{ij}}\right)L_{\mathrm{angle}}=\mathrm{MSE}\left({∠}_{\mathrm{ijk}},{\mathrm{∠̂}}_{\mathrm{ijk}}\right)L_{\mathrm{dihedral}}={\left(\Delta_{\mathrm{periodic}}\left(\tau_{\mathrm{ijkl}},{\mathrm{\tau ̂}}_{\mathrm{ijkl}}\right)\right)}^{2}$$

where *d*_*ij*_, *d̂*_*ij*_ = bond length between atoms *i* and *j* with true/predicted coordinates; ∠_*ijk*_, ∠̂_*ijk*_ = interior angle between bonded atoms *i*–*j*–*k* with true/predicted coordinates; τ_*ijkl*_, τ̂_*ijkl*_ = dihedral angle of atoms *i*-*j*-*k*-*l* with true/predicted coordinates; Δ_periodic_ = periodic difference within 0–2π radians, and *N* = number of atoms.

The inclusion of the structural loss is to help the VAE optimize to decode structures that are structurally similar to the target, even if the positions may be slightly different than the target. Similar approaches for enforcing local atomic relations are utilized in works concerning biological polymer structures.^21,36^ Final model hyperparameters are provided in Supporting Information C.

### Diffusion Transformer

Now that a joint latent space is learned, we require a way to find suitable structures for unseen polymer chemistries. We use a DiT architecture for our generative model M , operating within the latent space Z learned by the VAE. We use a similar DiT architecture as ADiT,^30^ however the previous work uses sinusoidal positional encodings for atomic tokens, which make it difficult for unordered atomic representations. In our case, we want to preserve the permutation invariant qualities of a GNN, so we use the positional encoded features and interactions in *C*_*i*_ to provide positional information relative to other atoms in the system. We also modify the attention mechanism to add a bias toward bonds, elaborated in the.

Our denoiser is implemented through a Gaussian flow matching approach, which is equivalent to denoising diffusion as one can be derived from the other.^30,37^ We start by encoding a DFT optimized structure into the latent space, using eq 5, to get $Z_{1}$ . Similar to other latent diffusion/flow matching works we denoise from zero-centered random noise $Z_{0}∼N\left(0,1\right)$ at *t* = 0 to $Z_{1}$ at *t* = 1.^30^ To train the transformer, we provide it with an interpolated sample $Z_{t}$ at a random time step t∼U(0,1) ,

8 $$Z_{t}=\left(1-t\right)Z_{0}+tZ_{1}$$

We can pose the learning problem as the linear ordinary differential equation (ODE),

9 $$u_{t}=Ż\left(Z_{t}\right)=\frac{Z_{1}-Z_{t}}{1-t}$$

The final prediction task is defined as,

10 $${Ẑ}_{1}=M\left(Z_{t},Z_{\mathrm{sc}},t,C_{i},S\right),{û}_{t}=\frac{{Ẑ}_{1}-Z_{t}}{1-t}$$

where *S* is learned embedding that represents the data set used during generation by projecting a one-hot encoding of the data set index into the model dimension. This is used exclusively during joint training. Only the embedding for the polyChainStructures data set is used during inference. $Z_{\mathrm{sc}}$ denotes a self-conditioning input, which corresponds to a previous prediction of ${Ẑ}_{1}$ . Self-conditioning improves autoregressive molecular generation.^30,38^ Our training uses a two-pass approach: first predicting ${Ẑ}_{1}$ with $Z_{\mathrm{sc}}=⌀$ , then feeding this prediction back as $Z_{\mathrm{sc}}$ for refinement. To avoid overreliance, we randomly drop self-conditioning ( $Z_{\mathrm{sc}}=⌀$ ) with probability 0.5 during training. Within M , the latent features and conditioning signals are incorporated as

$$\mathrm{x̃}=E_{Z}\left([Z_{t};Z_{\mathrm{sc}}]\right)+C_{i},c=d+E_{t}\left(t\right)$$

where *x̃* is input and *c* is the modulation conditioning for attention blocks from.^39^ $E_{Z}$ and *E*_*t*_ are embedding blocks.

The training objective is defined as the atom-wise mean squared error between ${\hat{u_{t}\;}}^{\left(i\right)}$ and *u*_*t*_^(*i*)^ for *N* atoms in a system:

11 LG=1N∑i=1N∥ut(i)−ût(i)∥2=1N∑i=1N∥Z1(i)−Zt(i)1−t−Ẑ1(i)−Zt(i)1−t∥2=1(1−t)2·1N∑i=1N∥Z1(i)−Ẑ1(i)∥2

To prevent numerical instability in the loss function calculation, we clip the value of *t* to 0.9, in accordance with previous work.^30^

During inference, we sample an initial latent $Z_{0}∼N\left(0,I\right)$ and iteratively denoise it using *T* steps of Euler integration:

$$Z_{t+\Delta t}=Z_{t}+\Delta t{û}_{t}$$

where Δ*t* = 1.0/*T*. This process generates a final conformation ${Ẑ}_{1}$ , which is decoded into a 3D atomic system using D . We compare generation hyperparameters and their efficiency in Supporting Information B, and show final model hyperparameters in Supporting Information C.

#### Relative Positional Encoding Attention Modification

With a traditional attention mechanism, DiT is forced to learn bonding relationships from the atom-wise conditional embeddings. For smaller systems, this can provide enough information for proper structure prediction, but this may be a problem for larger systems. Alphafold3, which operates on larger atomic systems than traditional material structure generation models, utilizes a pairformer^21^ with a relative position encoding to allow tokens to attend to nearby neighbors by conditioning the attention weights of the diffusion module.

We employ a lightweight relative positional encoding with attention biasing mechanism in the polyGen DiT module to differentiate local atomic interactions from global. First, we encode pairwise relationships with a one-hot graph-distance tensor

$$D\in {\left\{0,1\right\}}^{N\times N\times 5}$$

where each of the five channels indicates whether atoms *i* and *j* are (1) identical, (2) bonded, (3) separated by an angle, (4) separated by a dihedral, and (5) beyond four bonds apart. An MLP *f*_*D*_ then maps each one-hot vector *D*_*i*,*j*_ to a scalar bias, which is added directly into the scaled dot-product attention:

12 $${\mathrm{bias}}_{i,j}=f_{D}\left(D_{i,j}\right)$$

13 $${a\mathrm{ttention}}_{i,j}=\mathrm{Softmax}\left(\frac{Q_{i}\cdot K_{j}}{\sqrt{\mathrm{num}\_\mathrm{heads}}}+{\mathrm{bias}}_{i,j}\right)$$

Intuitively, nearby atoms (bonds, angles, dihedrals) should have a higher bias and other atoms should have a lower bias. With a per-DiT-block learnable bias, the DiT has the ability to allocate more attention weight while still retaining the ability to attend globally in some layers.

### Post-Generation Filtering

Often, sampling from a flow matching or diffusion model can lead to unphysical generations. In Figure 2c, we show postgeneration filtering as a sanity check of generation. First, we take the final predicted system (atom_type_*i*_, *b̂*_*z*_, *f̂*_*i*_) and use the Cartesian distances between atoms to calculate the predicted structure, Ĝ=(V,Ê) . If the ground truth graph G and the predicted graph Ĝ are isomorphic, i.e., whether G≅Ĝ , then it passes the filter. If any bond length is <0.8 Å then the sample is filtered out. Samples that pass this filtering criteria will be “successful” and those that do not are “unsuccessful.” However, a successful prediction of connectivity does not guarantee an accurate 3D structure, and the successful samples are evaluated against the optimized 3D structures in the Results and Discussion section.

### Machine Learning Techniques

Our main polymer DFT data set, polyChainStructures (elaborated in the Data set and Evaluation Metrics section), and the QM9 data set are imbalanced, with the latter training set being ≈ 33x larger. To handle this we upsample the polyChainStructures data by 30× per epoch. During training for the polychain data set only, this upsampling ratio is held the same for comparison, so that both models see the same amount of polyChainStructures data per epoch. To learn equivariance, we randomly rotate and translate the systems at every training step for both the VAE and the DiT.

The autoencoder models were trained for 300 epochs. The highest-performing validation checkpoint was taken. The diffusion models were trained for a maximum of 500 epochs. Training time took approximately 140 h on 2 L40S GPUs, with each epoch taking 17 min, and each parallel batch taking on average 0.112 s.

## Results and Discussion

### Data set and Evaluation Metrics

The main data set, polyChainStructures, consists of 3855 DFT-optimized infinite polymer chain structures, with a maximum of 208 atoms, including hydrogens. This structures data set was used to calculate the electronic bandgap (*E*_*g*_) of the polymers.^13^ Further details of this already open-sourced data set, as well as DFT methods can be found in.^12^ We use a 3084/386/385 (train/validation/test) split for the polyChainStructures data set. The QM9 data set, which contains DFT-optimized small-molecule conformations, is used to augment the training, allowing the models to learn local patterns from molecular structures. We use the train set from QM9 ^25^ which is a size of 100 K molecules. We acknowledge the presence of larger molecular data sets (i.e., GEOM) that could be used to further augment our model’s learning.^40^ However, from a structural perspective, the conformational behavior of nonzero temperature molecules found in GEOM differs significantly from the idealized infinite chains at 0K modeled in this work.

We benchmark polymer structure prediction on our polyChainStructures test set with our method. Unlike crystals, polymers are amorphous and can adopt many valid low-energy conformations, making conventional structure matching^41^ unsuitable. Molecule generation is evaluated with root mean squared distance (RMSD) after rotational alignment,^24,42^ but this does not account for conformational variability experienced by infinite chains, where chains can have a high point-by-point RMSD but have a structural match. In our case, we find that the distribution RMSDs of successfully generated samples from the ground truth can be a measure of the conformational diversity. We align the chains before calculating the periodic RMSD.

To this end, we compare the predicted conformations against the DFT-optimized structures by examining distributions over bond lengths, bond angles, and dihedrals. We quantify similarity using the forward Kullback–Leibler (KL) divergence from the predicted distribution to the ground truth. The task is to generate a distribution of plausible polymer conformations and assess the likelihood that the DFT-optimized structure could have been drawn from this predicted distribution. For a set of predicted quantities *Q*_pred_ we match it to the set of DFT predicted quantities *Q*_DFT_ with

14 $$L_{\mathrm{KL}}=D_{\mathrm{KL}}\left(Q_{\mathrm{DFT}}∥Q_{\mathrm{pred}}\right)=\int Q_{\mathrm{DFT}}\left(z\right)\log\frac{Q_{\mathrm{DFT}}\left(z\right)}{Q_{\mathrm{pred}}\left(z\right)}dz$$

In practice, we do this over a discrete set of buckets where d*z* is 0.001 of the predefined ranges of the quantities, which could be bond lengths (0.9 to 2.0), angles (0 to 180), or dihedrals (0 to 360).

In order to evaluate the veracity of the generated structures, given that that they were conditioned on Density Functional Theory (DFT) based training structural data, we have adopted the following procedure. To estimate the energies of the generated structures, we performed first-principles DFT calculations using the Vienna *Ab initio* Simulation Package (VASP).^43,44^ Projector-augmented wave (PAW)^45,46^ pseudopotentials were used to include electron–ion interactions within the generalized gradient approximation (GGA). Weak van der Waals interactions are incorporated by applying the DFT-D3 method,^47^ along with energy convergence criteria for the self-consistent electronic loop, set to 10^–5^ eV. A Gaussian smearing has been used with a smearing width of 0.01 eV. The kinetic energy cutoff was set to 520 eV, 30% higher than the default maximum value given in the pseudopotential to ensure better convergence and accuracy. The Brillouin zone is sampled using 1 × 1 × 1, Γ-centered k-grid.

### Structure Matching Results

We find that our model is capable of predicting polymer structures that closely match the linear chain structure calculated by DFT. To provide context to our rationale, Figure 3 shows examples of predicted polymers compared to the ground truth of the data set. In Figure 3a–d we see the capabilities of generating systems with qualitatively similar structures after filtering. We also provide examples of errors that are caught by our filtering. In Figure 3a,c, we see examples of carbon atoms incorrectly predicted within a supposedly aromatic ring in the backbone, which lead to incorrect connectivity. Many failed structures, especially the one in Figure 3b, can easily be fixed with an energy minimization or a heuristic-based increasing of the C–H bond, demonstrating the efficacy of the remaining generation and the stringent nature of the generation filter. Also, the complexity of these structures should be noted, given chain size, branching, and the number of rings present.

**Figure 3:**
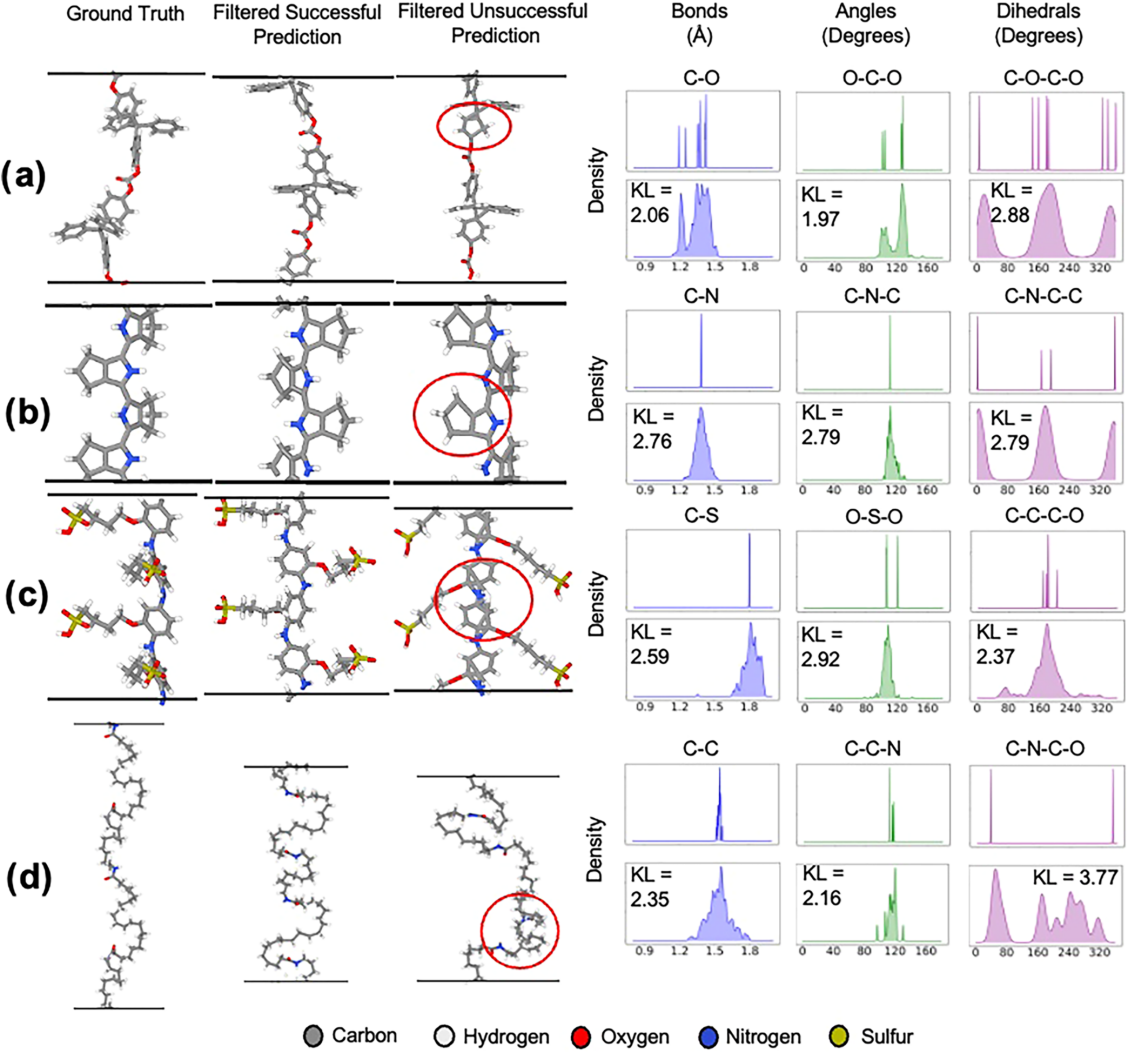
Visual samples from 100 generations per polymer type. Each row (a–d) displays examples from a single polymer. The ground truth, a successfully generated sample, and an unsuccessful sample are provided. The unsuccessful samples highlight the errors that caused the incorrect connectivity. Note that the structures for (b) and (c) are replicated along the *z*-axis for visual clarity of the structure. The visualizations are accompanied by corresponding ground truth distributions (top) and model-predicted distributions (bottom) of molecular bonds, angles, and dihedrals for that polymer..

To quantify structural similarity, we compare bond length, triplet angle, and dihedral angle distributions for some atomic species. Predicted structures should exhibit peaks comparable to optimized structures. The forward KL divergences establish benchmarks for acceptable “matches” in bond lengths, angles, and dihedrals.

We find that our approach can find relative trends in the majority of bond lengths, but lacks precision. For example, the predicted C–O bonds in Figure 3a shows 2 peaks for single and double bonds, with noisy predictions scattered by at least 0.1 Å around these peaks. In Figure 3b,c, both predicted C–N and C–S bond distributions show peaks corresponding with the true bond length, but with wide distributions. Generation is precise to the order of Å, but not on the scale of picometers. Precision on the level of picometers will be necessary to properly distinguish between bond types before our approach is scaled to larger system sizes.

We find that the distribution peaks of angles and dihedrals match the ground truth peaks, especially the O–C–O and O–S–O bonds in Figure 3a,c, respectively. The dihedral distributions can be used to validate the prediction of correct local structures in the polymer. For example, the C–N–C-C dihedrals in Figure 3b validate the feasible prediction of the nitrogen containing 5-membered ring in the backbone. In Figure 3c, the C–C–C-O dihedral, located on a branch, shares a peak with the ground truth at 160° but has a few predictions around 80° and 320°. This reflects the difficulties in generating branched structures, which may have conformational variability.

Figure 3d shows predictions for the largest system in the test data set, containing 208 atoms. Only 3 out of 100 generated structures pass the filtering when using position-biased attention, while the DiT model with vanilla attention fails to produce any valid structure. The broad distribution of predicted C–N–C–O dihedral angles and their high deviation from the ground truth further highlight the lack of conformational viability. These results underscore a clear limitation in handling larger systems, likely due to constraints in the training data set, and illustrate the challenges of generating feasible structures for complex polymer repeat units.

### Prediction of Diverse Samples

As highlighted earlier, polymer structures do not reside in one fixed conformation but occur as an ensemble of minimized conformations, even close to 0 K. Our training data set contains only 1 conformational example out of the potential ones, because the generation of these ensembles is costly. A useful generation model would produce a diverse ensemble of low-energy state conformations. In Figure 4a, despite the lack of per-polymer distribution in our data set, polyGen can generate an ensemble of diverse structures with variable repeat unit lengths. Because of the Gaussian flow matching approach, the initial Gaussian sample results in the sampling of different trajectories by the DiT, and therefore different points are generated in the latent space. Figure 4b shows a clear Gaussian-like distribution of generated conformations for the polymer in Figure 4a, as a measure of RMSD from the ground truth. Across the full test set, the standard deviation of this distribution for polyGen indicates a wider variety of conformations per generation compared to PSP. In contrast, PSP has a maximum standard deviation of 1. Because it is a physics- and heuristic-based framework, it prioritizes correct connectivity and local energy minimization, making it less suited for diverse generation.

**Figure 4:**
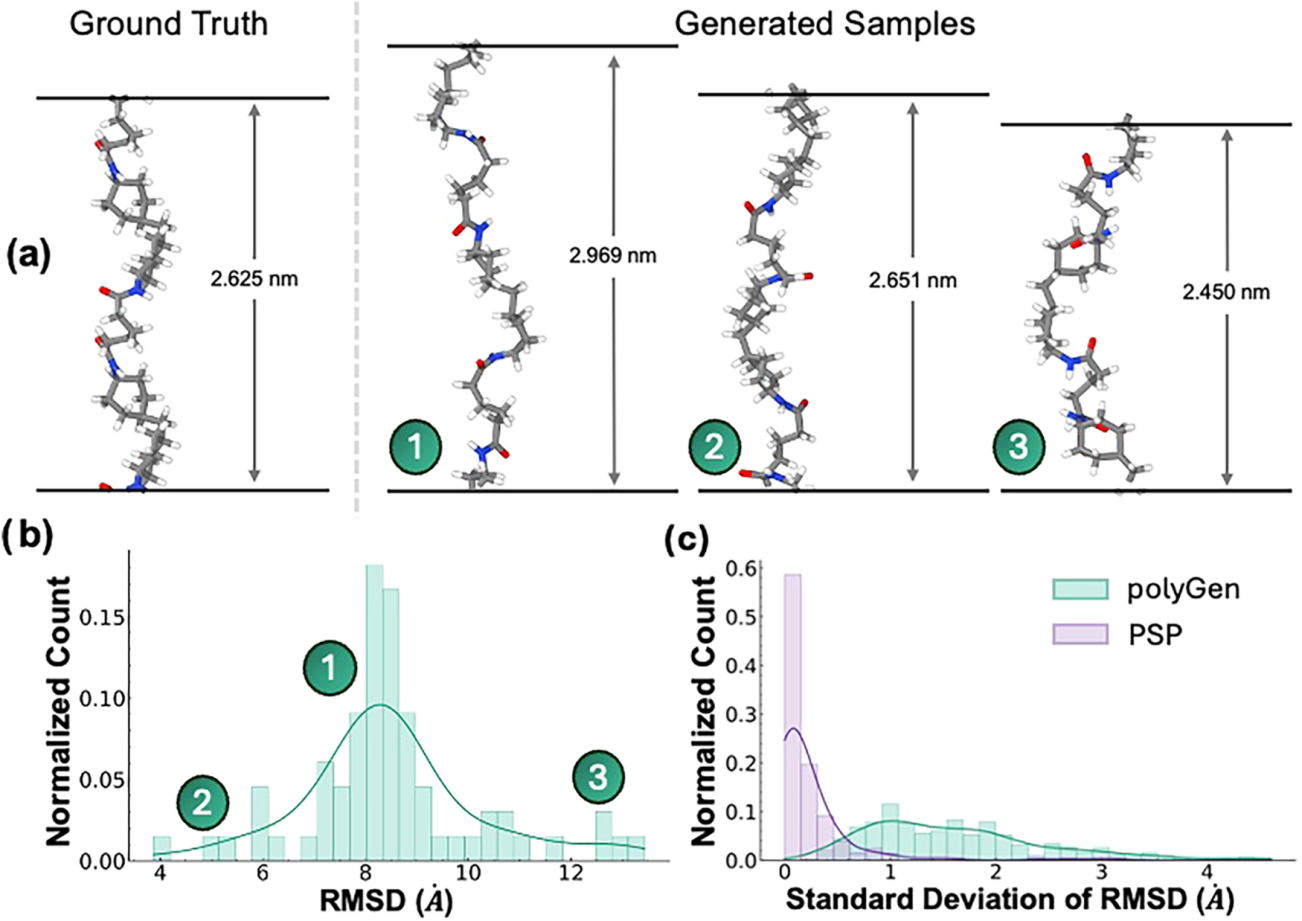
(a) Demonstration of diverse structures generated from polyGen for the same repeat unit, compared to the ground truth structure. (b) The distribution of RMSD from the ground truth of the generated polymers in (a) demonstrates the variety of conformations that were generated. (c) The standard deviations of the generated polyGen samples for each test polymer..

### Overall Structural Results

We see that joint training on multiple DFT data sets can improve model performance, in accordance with previous studies.^16,30^ In this section, we show the specific areas in which the inclusion of the QM9 data set can benefit the generation of polymer structures by comparing the joint data set approach with a model fully trained on just the polyChainStructures data set. Figure 5 shows the overall KL divergences for bond lengths, angle lengths, and dihedrals, and comparison of predicted z-height. The inclusion of the QM9 data set improves predictions on the local levels of polymer chains, as the KL divergences of bonds and angles decrease by 41.0 and 29.0%, respectively, upon the addition of the QM9 data set. Larger features, such as dihedral angles and the z-height show limited improvement, with dihedrals improving by 16.1%. This is likely because small molecules do not provide any insight into the macrostructural properties of polymers. Overall, we find that 36.1% of the generated structures by the jointly trained model pass our filtering, whereas 27.4% samples are generated correctly when using only the polyChainStructures data set for training.

**Figure 5:**
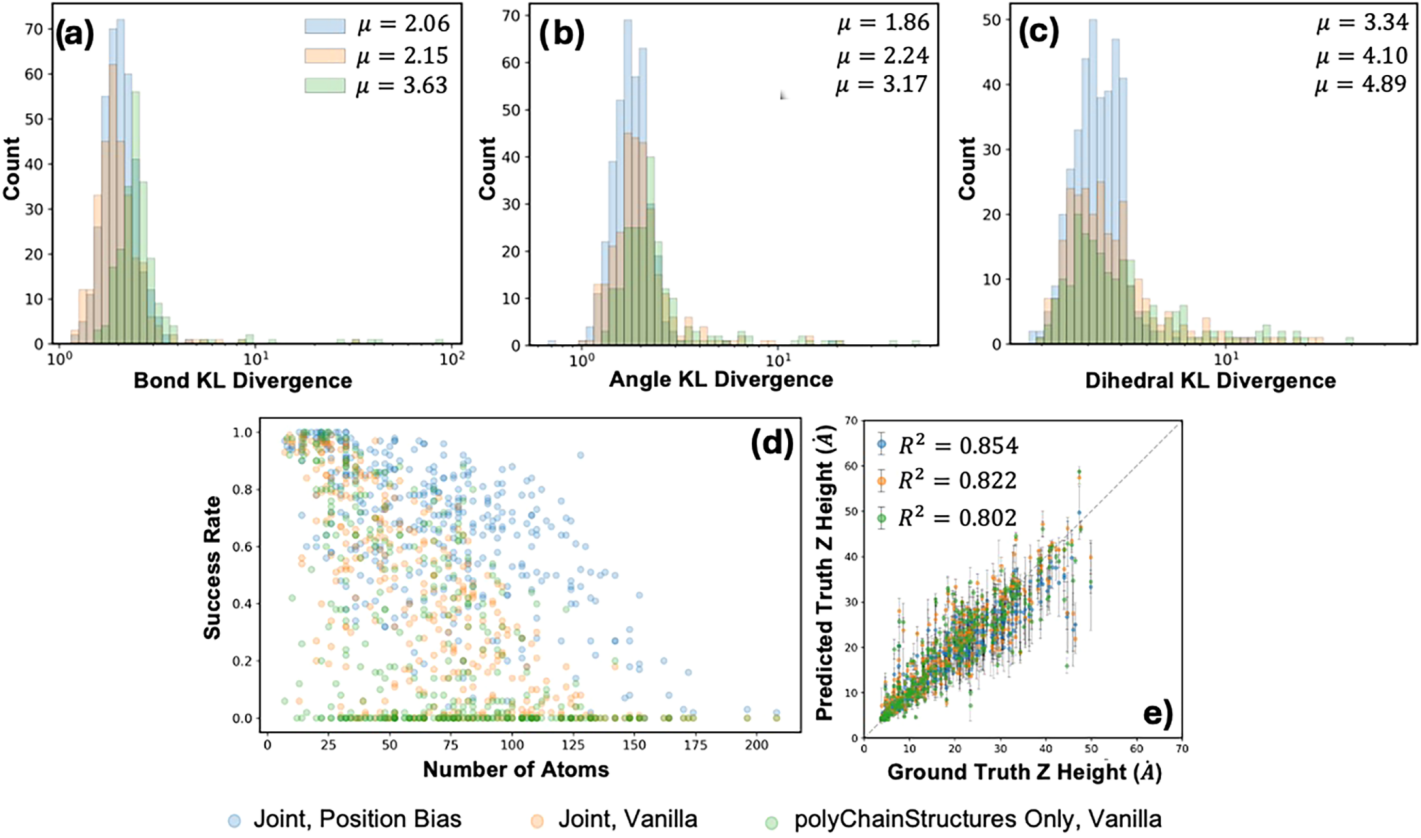
KL Divergences of Joint training for Position Bias, Joint Training and polyChainStructures Only for (a) Bonds (b) Angles and (c) Dihedrals. (d) The comparison of z-height prediction vs the DFT ground truth. (e) The success rate of filtering as a function of the atom count of the system.

Another major improvement is seen when including position attention biasing in the DiT module. Because local interactions are explicitly specified, we find that the positional encoding improves generation success from 36.9 to 64.8% when looking at isomorphism of the predicted structure compared to the original graph. Figure 5 showcases the improvement of polyGen with a relative encoding bias on all metrics, notably the decrease of angle and dihedral KL Divergences by 33.5 and 21.2%, respectively.

As highlighted in Figures 4 and 5e, we expect some variability in the prediction of the z-height of the polymer chain. Despite this, we can achieve an *r* ^2^ score of 0.854 between the predicted and DFT heights, meaning that the model can differentiate between dense vs sparsely packed structures. Additionally, we see a larger standard deviation and error in predictions for larger repeat units (>10 Å), because these repeat units may show more flexibility. Figure 5d also highlights the difficulty of generation as system size increases. Most generated systems with <25 atoms show success rates >0.5. For system sizes greater than 150 atoms, the model with vanilla attention cannot generate any systems with proper connectivity, but the relative position bias substantially improves this.

Intuitively, it would also seem that complex polymeric structures would be more difficult to generate. To this end, we also compare these metrics with the Synthetic Accessibility (SA) in Supporting Information A. We see loose correlations with KL divergences of bond length, angles, and dihedrals with the SA score, but the ratio of successful generations of a polymer do not have a strong correlation with the SA score. Therefore, generation success could be independent from SA score and more reliant on the system size.

The speed of polyGen of the successful structural generations is compared with PSP. polyGen generated samples in batches of 200, at an overall average of about 0.308 s per successful sample, taking advantage of the parallelizability of batched evaluations with transformers. PSP generated the samples at a rate of about 30.2 s per successful sample.

### Energetic Results

Figure 6 shows the difference in total potential energies of the polyGen and PSP samples when compared with DFT. Both methods show low energy differences, i.e., both produce structures close to the DFT ground state. PSP, being a physics-based optimizer, produces structures with total potential energy less than what is generated by polyGen. 53.8% of polyGen samples and 74.7% of PSP samples are within 0.1 eV/Atom of DFT. We note that polyGen has one sample (0.26%) and PSP has 6 (1.6%) generated samples that are >1.0 eV/Atom from DFT. PSP tends to generate geometries close to the DFT ground state but does produce some large outliers, whereas polyGen produces slightly higher energies but with greater consistency, as demonstrated by a tighter distribution of energy differences with respect to DFT.

**Figure 6:**
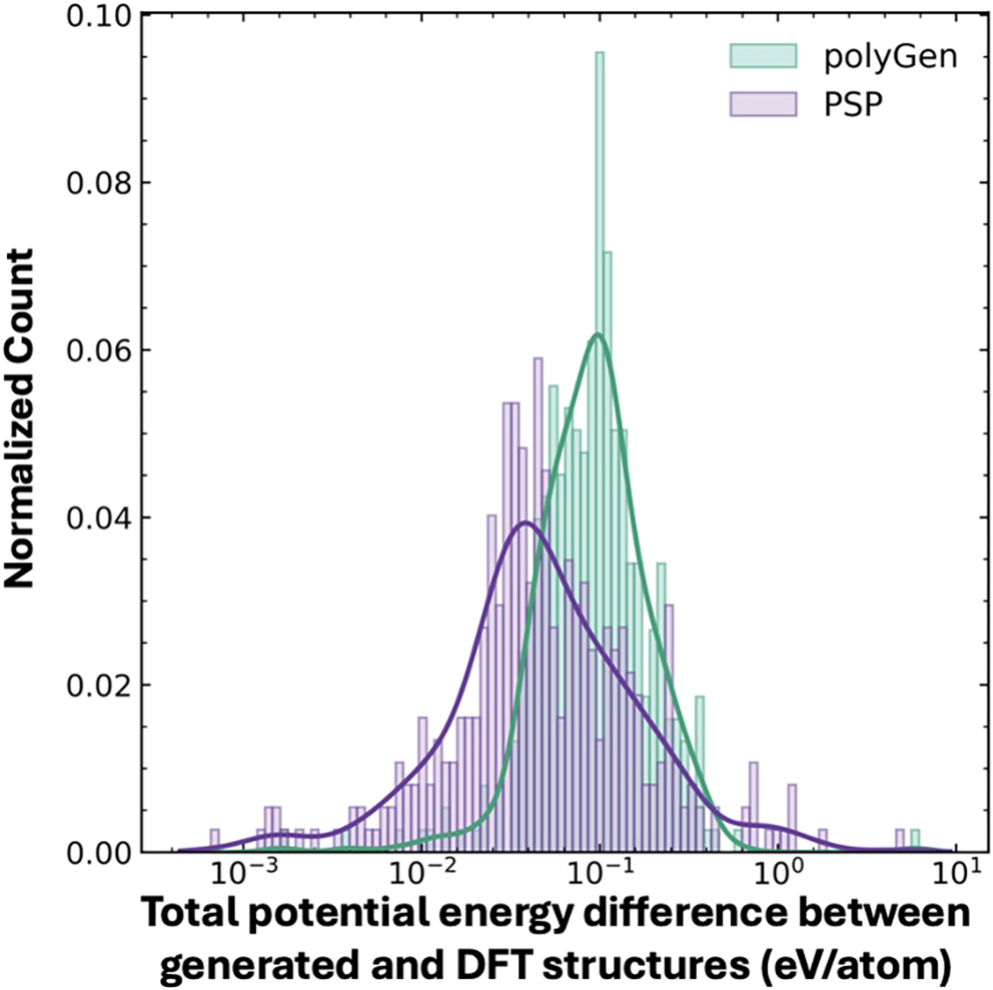
Difference in potential energy (eV/Atom) from polyGen and PSP to the DFT relaxed structures in the test set.

## Conclusions

In this study, we introduce a solution to the problem of atomic-level polymer structure generation—given only the atomic connectivity of a repeat unit (i.e., SMILES), polyGen can generate an ensemble of realistic 3D structures of synthetic polymers. Results demonstrate that polyGen effectively generates structures with bond lengths, angles, and dihedral distributions that align well with ground truth structures, and the quality of structures improves with the inclusion of positional biasing to the attention mechanism of the diffusion transformer. The model also successfully generates valid conformations for complex features like aromatic backbones and branches while correlating repeat unit chemistry with structural properties, such as repeat unit length. These initial results are particularly promising considering our polymer structure data set optimized with Density Functional Theory contains only 3855 systems. We also investigate the limitations and demonstrate the need for stringent benchmarking of future polymer structure generation techniques. While capturing relative trends in structure, the model lacks precision at the picometer scale needed to distinguish between bond types. When compared to a physics-based predictor, Polymer Structure Predictor, polyGen generates samples with slightly higher total potential energy compared to DFT, but with fewer outliers, greater speed, more conformational diversity, and more consistency. The current data set is a limiting factor for polyGen’s capabilities. Performance degrades significantly for larger polymeric systems, due to the lack of these in the data set. The data set also needs to include nonlinear network, ladder, larger branched structures, and conformations at nonzero temperatures. These will be the subject of future enquiry.

Given the successes of this proof-of-concept, future work will focus on expanding the training data set to include larger polymer systems, incorporating additional physics-informed constraints, and exploring hybrid approaches that combine latent diffusion with molecular dynamics simulations. polyGen represents an initial technique in the sparsely explored space of polymer structure generation, and addressing these challenges could transform it into an invaluable tool for computational materials science, accelerating the discovery of novel polymeric materials across numerous applications.

## Supplementary Material



## Data Availability

The data set used in this study is available on the Ramprasad group’s computational knowledge-base, Khazana (https://khazana.gatech.edu/dataset).

## Notes

Additional experiments, results, and minor implementation details (PDF)

## References

[1] Tran H., Gurnani R., Kim C., Pilania G., Kwon H.-K., Lively R. P., Ramprasad R. (2024). Design of functional and sustainable
polymers assisted by artificial intelligence. Nat. Rev. Mater..

[2] Batra R., Song L., Ramprasad R. (2021). Emerging materials
intelligence ecosystems
propelled by machine learning. Nat. Rev. Mater..

[3] Shukla S. S., Kuenneth C., Ramprasad R. (2024). Polymer informatics
beyond homopolymers. MRS Bull..

[4] Gurnani R., Shukla S., Kamal D., Wu C., Hao J., Kuenneth C., Aklujkar P., Khomane A., Daniels R., Deshmukh A. A. (2024). AI-assisted discovery
of high-temperature dielectrics
for energy storage. Nat. Commun..

[5] Phan B. K., Shen K.-H., Gurnani R., Tran H., Lively R., Ramprasad R. (2024). Gas permeability, diffusivity, and
solubility in polymers:
Simulation-experiment data fusion and multi-task machine learning. npj Comput. Mater..

[6] Chen L., Pilania G., Batra R., Huan T. D., Kim C., Kuenneth C., Ramprasad R. (2021). Polymer informatics:
Current status
and critical next steps. Mater. Sci. Eng., R.

[7] Doan
Tran H., Kim C., Chen L., Chandrasekaran A., Batra R., Venkatram S., Kamal D., Lightstone J. P., Gurnani R., Shetty P. (2020). Machine-learning predictions
of polymer properties with Polymer Genome. J.
Appl. Phys..

[8] Gurnani R., Kuenneth C., Toland A., Ramprasad R. (2023). Polymer Informatics
at Scale with Multitask Graph Neural Networks. Chem. Mater..

[9] Kern J., Su Y.-L., Gutekunst W., Ramprasad R. (2025). An informatics
framework for the design of sustainable, chemically recyclable, synthetically
accessible, and durable polymers. npj Comput.
Mater..

[10] Kern J., Chen L., Kim C., Ramprasad R. (2021). Design of
polymers for energy storage capacitors using machine learning and
evolutionary algorithms. J. Mater. Sci..

[11] Batra R., Dai H., Huan T. D., Chen L., Kim C., Gutekunst W. R., Song L., Ramprasad R. (2020). Polymers for extreme conditions designed
using syntax-directed variational autoencoders. Chem. Mater..

[12] Huan T. D., Ramprasad R. (2020). Polymer structure prediction from first principles. J. Phys. Chem. Lett..

[13] Kamal D., Tran H., Kim C., Wang Y., Chen L., Cao Y., Joseph V. R., Ramprasad R. (2021). Novel high
voltage polymer insulators
using computational and data-driven techniques. J. Chem. Phys..

[14] Yang L., Zhang Z., Song Y., Hong S., Xu R., Zhao Y., Zhang W., Cui B., Yang M.-H. (2024). Diffusion
models: A comprehensive survey of methods and applications. ACM Comput. Surv..

[15] Lipman, Y. ; Chen, R. T. ; Ben-Hamu, H. ; Nickel, M. ; Le, M. Flow Matching for Generative Modeling. 11th International Conference on Learning Representations, ICLR, Vol. 2023, 2023.

[16] Tran R., Lan J., Shuaibi M., Wood B. M., Goyal S., Das A., Heras-Domingo J., Kolluru A., Rizvi A., Shoghi N. (2023). The Open
Catalyst 2022 (OC22) dataset and challenges for oxide electrocatalysts. ACS Catal..

[17] Jumper J., Evans R., Pritzel A., Green T., Figurnov M., Ronneberger O., Tunyasuvunakool K., Bates R., Žídek A., Potapenko A. (2021). Highly accurate protein structure prediction
with AlphaFold. Nature.

[18] Jiao R., Huang W., Lin P., Han J., Chen P., Lu Y., Liu Y. (2023). Crystal structure prediction
by joint equivariant diffusion. Adv. Neural
Inf. Process. Syst..

[19] Xie, T. ; Fu, X. ; Ganea, O.-E. ; Barzilay, R. ; Jaakkola, T. S. Crystal Diffusion Variational Autoencoder for Periodic Material Generation. In International Conference on Learning Representations.

[20] Miller, B. K. ; Chen, R. T. ; Sriram, A. ; Wood, B. M. Flowmm: Generating materials with riemannian flow matching. In Forty-first International Conference on Machine Learning, 2024.

[21] Abramson J., Adler J., Dunger J., Evans R., Green T., Pritzel A., Ronneberger O., Willmore L., Ballard A. J., Bambrick J. (2024). Accurate
structure prediction of biomolecular
interactions with AlphaFold 3. Nature.

[22] Wohlwend J., Corso G., Passaro S., Reveiz M., Leidal K., Swiderski W., Portnoi T., Chinn I., Silterra J., Jaakkola T. (2025). Boltz-1: Democratizing Biomolecular Interaction
Modeling. bioRxiv.

[23] Hassan M., Shenoy N., Lee J., Stärk H., Thaler S., Beaini D. (2024). ET-Flow: Equivariant
Flow-Matching
for Molecular Conformer Generation. Adv. Neural
Inf. Process. Syst..

[24] Xu, M. ; Powers, A. S. ; Dror, R. O. ; Ermon, S. ; Leskovec, J. Geometric latent diffusion models for 3d molecule generation. In International Conference on Machine Learning, 2023; pp 38592–38610.

[25] Wu Z., Ramsundar B., Feinberg E. N., Gomes J., Geniesse C., Pappu A. S., Leswing K., Pande V. (2018). MoleculeNet: a benchmark
for molecular machine learning. Chem. Sci..

[26] Corso, G. ; Stärk, H. ; Jing, B. ; Barzilay, R. ; Jaakkola, T. S. DiffDock: Diffusion Steps, Twists, and Turns for Molecular Docking. In Eleventh International Conference on Learning Representations, 2023.

[27] Wang, F. ; Guo, W. ; Ou, Q. ; Wang, H. ; Lin, H. ; Xu, H. ; Gao, Z. PolyConf: Unlocking Polymer Conformation Generation through Hierarchical Generative Models. In Forty-second International Conference on Machine Learning, 2025.

[28] Afzal M. A. F., Browning A. R., Goldberg A., Halls M. D., Gavartin J. L., Morisato T., Hughes T. F., Giesen D. J., Goose J. E. (2021). High-throughput
molecular dynamics simulations and validation of thermophysical properties
of polymers for various applications. ACS Appl.
Polym. Mater..

[29] Orselly M., Devemy J., Bouvet-Marchand A., Dequidt A., Loubat C., Malfreyt P. (2022). Molecular simulations of thermomechanical properties
of epoxy-amine resins. ACS Omega.

[30] Joshi, C. K. ; Fu, X. ; Liao, Y.-L. ; Gharakhanyan, V. ; Miller, B. K. ; Sriram, A. ; Ulissi, Z. W. All-atom Diffusion Transformers: Unified generative modelling of molecules and materials. In Forty-second International Conference on Machine Learning, 2025.

[31] Battaglia P., Pascanu R., Lai M., Jimenez Rezende D. (2016). Interaction networks
for learning about objects, relations and physics. Adv. Neural Inf. Process. Syst..

[32] Dwivedi, V. P. ; Luu, A. T. ; Laurent, T. ; Bengio, Y. ; Bresson, X. Graph Neural Networks with Learnable Structural and Positional Representations. In International Conference on Learning Representations, 2022.

[33] Dwivedi V.
P., Joshi C. K., Luu A. T., Laurent T., Bengio Y., Bresson X. (2023). Benchmarking
graph neural networks. J. Mach. Learn. Res..

[34] Wang, Y. ; Elhag, A. A. A. ; Jaitly, N. ; Susskind, J. M. ; Bautista, M. A. Swallowing the Bitter Pill: Simplified Scalable Conformer Generation. In International Conference on Machine Learning, 2023.

[35] Min, E. ; Chen, R. ; Bian, Y. ; Xu, T. ; Zhao, K. ; Huang, W. ; Zhao, P. ; Huang, J. ; Ananiadou, S. ; Rong, Y. Transformer for graphs: An overview from architecture perspective. arXiv:2202.08455. arXiv.org e-Print archive. https://arxiv.org/abs/2202.08455, 2022.

[36] Mariani V., Biasini M., Barbato A., Schwede T. (2013). lDDT: a local
superposition-free
score for comparing protein structures and models using distance difference
tests. Bioinformatics.

[37] Gao, R. ; Hoogeboom, E. ; Heek, J. ; Bortoli, V. D. ; Murphy, K. P. ; Salimans, T. Diffusion Meets Flow Matching: Two Sides of the Same Coin, 2024; https://diffusionflow.github.io/.

[38] Stärk, H. ; Jing, B. ; Barzilay, R. ; Jaakkola, T. S. Harmonic Self-Conditioned Flow Matching for Multi-Ligand Docking and Binding Site Design CoRR arXiv:2310.05764. arXiv.org e-Print archive. https://arxiv.org/abs/2310.05764, 2023.

[39] Peebles, W. ; Xie, S. Scalable diffusion models with transformers. In Proceedings of the IEEE/CVF International Conference on Computer Vision, 2023; pp 4195–4205.

[40] Axelrod S., Gomez-Bombarelli R. (2022). GEOM, energy-annotated molecular conformations for
property prediction and molecular generation. Sci. Data.

[41] Ong S. P., Richards W. D., Jain A., Hautier G., Kocher M., Cholia S., Gunter D., Chevrier V. L., Persson K. A., Ceder G. (2013). Python Materials Genomics
(pymatgen): A robust, open-source python
library for materials analysis. Comput. Mater.
Sci..

[42] Jing B., Corso G., Chang J., Barzilay R., Jaakkola T. (2022). Torsional
diffusion for molecular conformer generation. Adv. Neural Inf. Process. Syst..

[43] Kresse G., Furthmüller J. (1996). Efficient iterative schemes for ab
initio total-energy
calculations using a plane-wave basis set. Phys.
Rev. B.

[44] Kresse G., Furthmüller J. (1996). Efficiency
of ab-initio total energy calculations for
metals and semiconductors using a plane-wave basis set. Comput. Mater. Sci..

[45] Blöchl P. E. (1994). Projector
augmented-wave method. Phys. Rev. B.

[46] Kresse G., Joubert D. (1999). From ultrasoft pseudopotentials
to the projector augmented-wave
method. Phys. Rev. B.

[47] Grimme S., Antony J., Ehrlich S., Krieg H. (2010). A consistent
and accurate
ab initio parametrization of density functional dispersion correction
(DFT-D) for the 94 elements H-Pu. J. Chem. Phys..

