# Supplementary material for: A Learning Framework for Atomic-Level Polymer Structure Generation
Source: Chem Mater. 2025 Sep 11;37(18):7337–46. doi: 10.1021/acs.chemmater.5c01644 (PMC12461786; doi:10.1021/acs.chemmater.5c01644)
Supplement: Supplementary file 1 [file cm5c01644_si_001.pdf]

A Learning Framework for Atomic-level Polymer Structure Generation

Ayush Jain,[†,‡] Ashutosh Srivastava,[†] and Rampi Ramprasad[*,†]

†*School of Materials Science and Engineering, Georgia Institute of Technology, 771 Ferst Drive Atlanta, GA 30332*

‡*School of Computational Science and Engineering, Georgia Institute of Technology, 756 W Peachtree St NW, Atlanta, GA 30332*

E-mail: rampi.ramprasad@mse.gatech.edu

A) Impact of SA Score on Generation

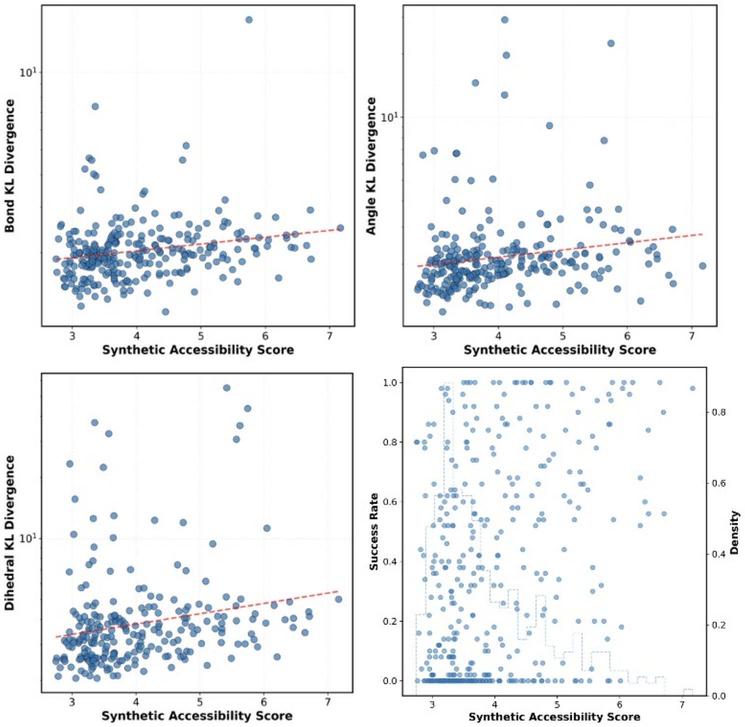

Figure 1: Impacts of Synthetic Accessability (SA) score[1] on the quality of generation bonds, angles, dihedrals, and successes after filtering. The SA score is calculated through RDKit.

B) Generation Tuning

We present additional experiments with the vanilla attention DiT determining the quality of generation across changes to generation hyperparameters. In Figure 3a we find that increasing the number of the integration steps increases the the quality of generation until ∼100 steps. In Figure 3b, the percentage of successful samples increases slightly with the number of generated conformations per polymer chain, reaching saturation at 100 samples, which is a practical generation limit given our computational constraints.

Several works for autoregressive generative models for ODE/SDE propose the addition of noise during the denoising process or ”churn” to improve the generation quality.[2,3] Intuitively, this approach slightly noises the latent at each integration step, and allows the model to correct for previous errors. The impact of this stochastic generation is unclear when it comes to an all-atom latent space. In this context, the additional noise could ”pull” the current trajectory out of an unfavorable area of the latent space. We experiment with varying

amounts of churn across the integration, where a higher churn value corresponds to more noise re-injected at each step. We find that a lower level of churn ≤ 0.1 is more beneficial to generation success in the all-atom latent space. We note that there may be additional tuning done, such as ranges of t steps to apply churn. The algorithm for stochastic generation is presented in Algorithm 1.

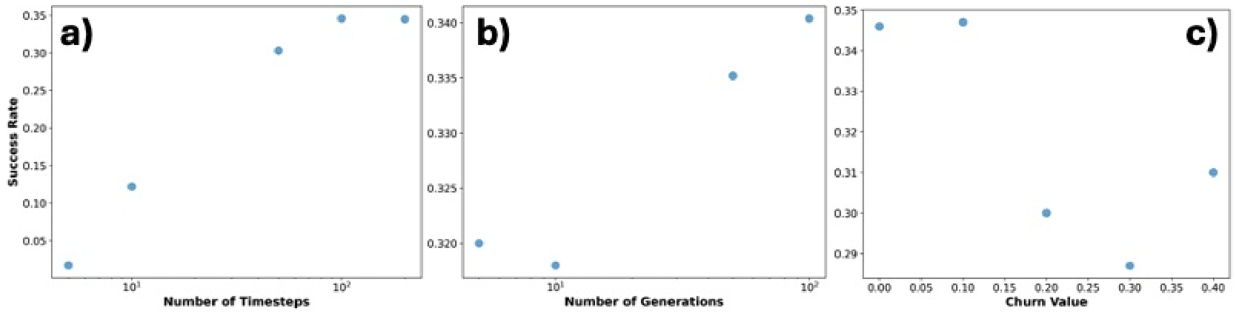

Figure 2: Generation filter success rate for a sample of 100 test polymers vs. a) number of the timesteps, b) number of generations per sample, c) level of churn.

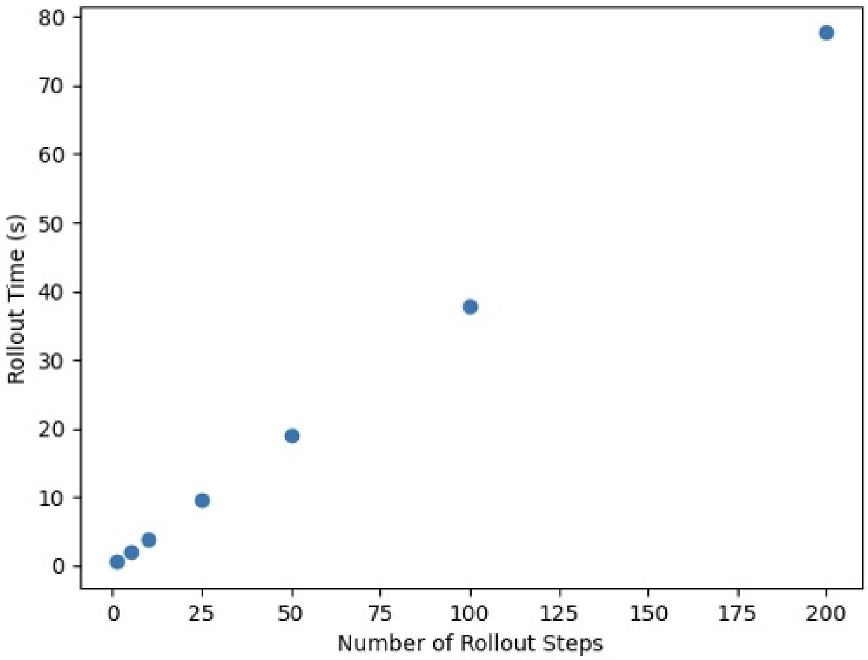

Figure 3: Generation time for different numbers of rollout steps.

The number of steps for rollout was chosen as a balance between compute time and accuracy. As shown in 3, the amount of time and cost scales linearly with rollout steps. We

choose 100 steps because the accuracy gains from increased rollouts plateaus as time still increases linearly.

Algorithm 1 PolyGen Generation with Stochasticity

Require: Polymer connectivity graph G, number of sampling steps T, stochasticity level churn

Conditional Encoding $C_i \leftarrow \mathcal{E}_c(G)$

Set $\Delta t \leftarrow \frac{1}{T}$

Set $\gamma \leftarrow \frac{\text{churn}}{T}$

Sample initial latent $\mathcal{Z}_0 \sim \mathcal{N}(0, I)$

Set $\mathcal{Z}_{sc} \leftarrow \emptyset$

for $n \leftarrow 0$ to $T - 1$ **do**

  Set $t \leftarrow \frac{n}{T}$

  Sample $\epsilon \sim \mathcal{N}(0, I)$

  $\Delta\hat{t} \leftarrow \gamma(1 - t)$

  $\hat{t} \leftarrow \max(t - \Delta\hat{t}, 0)$

  $\mathcal{Z}_t \leftarrow \mathcal{Z}_t + \Delta\hat{t}\sqrt{t^2 - \hat{t}^2}\epsilon$

  Predict $\hat{\mathcal{Z}}_1 = \mathcal{M}(\mathcal{Z}_t, \mathcal{Z}_{\text{sc}}, \hat{t}, C_i)$

  $\mathcal{Z}_{\text{sc}} \leftarrow \hat{\mathcal{Z}}_1$

  Compute velocity $\hat{u}_t = \frac{\hat{\mathcal{Z}}_1 - \mathcal{Z}_t}{1-t}$

  Update $\mathcal{Z}_{t+\Delta t} = \mathcal{Z}_t + \hat{u}_t \Delta t$

end for

Decode final structure: $\hat{f}, \hat{p}, \hat{b}_z = \mathcal{D}(\mathcal{Z}_1, C_i)$

C) Model Hyperparameters

When selecting model hyperparameters, we compared the effects of model size and relative bias inclusion. Using checkpoints from 150 training epochs, we evaluated success rates across different model configurations (Figure 4). We found that incorporating relative bias improved performance more than increasing the number of transformer layers, dimensions, and heads.

Table 1: Hyperparameters for Autoencoder Model

**Parameter**	**Value**
General Parameters	
Latent Dimension	8
Learning Rate	10^{-4}
Weight Decay	0.0
Architecture	
Token Embedding Dimension	512
Attention Heads	8
Feedforward NN Dimension	1024
Number of Layers	8
Loss Weights	
Loss Weight: Bounding Box (polychain)	1.0
Loss Weight: Bonds (polychain)	2.0
Loss Weight: Angles (polychain)	2.0
Loss Weight: Dihedrals (polychain)	1.0
Loss Weight: Fractional Coordinates (polychain)	10.0
Loss Weight: KL Divergence (polychain)	10^{-5}
Loss Weight: Position (qm9)	1.0

Table 2: Hyperparameters for Latent Diffusion Model

**Parameter**	**Value**
Architecture	
Latent Dimension	8
Token Embedding Dimension	512
Attention Heads	8
Number of Layers	12
Training	
Learning Rate	10^{-4}
Weight Decay	10^{-4}
Generation	
Generation Timesteps	100
Churn	0.0

Table 3: Hyperparameters for Latent Diffusion Model - Large (250 M)

Parameter	Value
Architecture	
Latent Dimension	8
Token Embedding Dimension	768
Attention Heads	16
Number of Layers	24
Training	
Learning Rate	10^{-4}
Weight Decay	10^{-4}
Generation	
Generation Timesteps	100
Churn	0.0

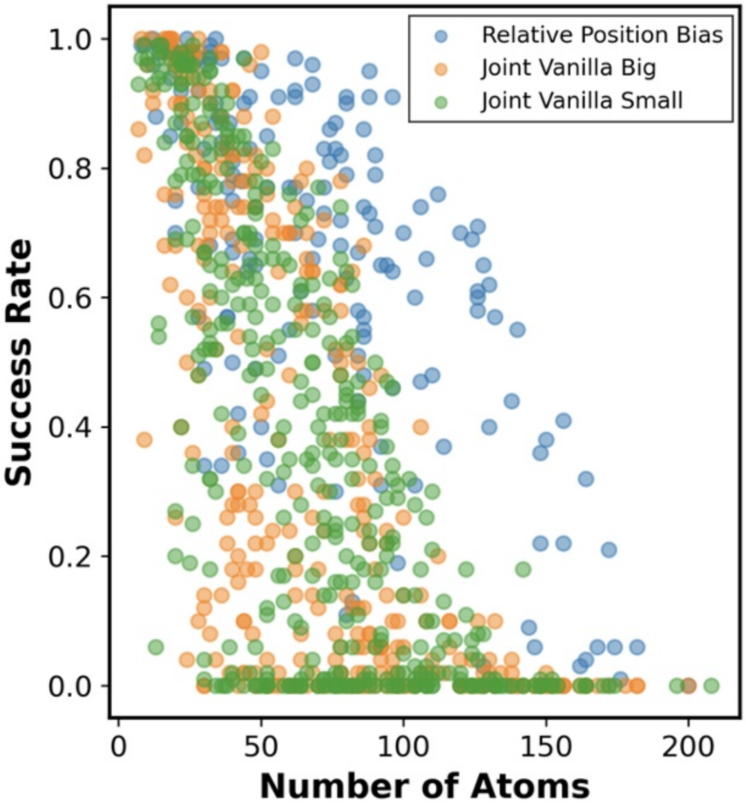

Figure 4: Comparison of successful generations on the test set between The LDM model (Joint Vanilla Small), LDM-Large (Joint Vanilla Big), and the LDM with Relative bias (Relative Position Bias).

The latent space dimension was fixed at 8, consistent with previous DiT work[4] that demonstrated optimal performance at this size. While larger latent spaces could potentially improve performance, they may not provide the desired compression given the information

encoded (fractional coordinates, Cartesian positions, z box length, and atomic connectivity). Additionally, larger latent dimensions would require substantially more training data to be effective and would reduce the compression efficiency of the model."

D) FlowMM Comparison

Polymer structure prediction presents unique challenges, making off-the-shelf models unsuitable as direct benchmarks. To underscore the need for polymer-specific models, we adapted FlowMM[5]—a Riemannian flow-matching framework for crystal structures using the equivariant DiffCSP architecture.[6] The key modification that was needed for FlowMM was bonding information of the polymer. This was done by adding an additional edge type upon the creation of the radius graph: edge type 0 are non-bonded interactions and edge type 1 is a bonded interaction.

FlowMM operates on the product manifold

$$\mathcal{M}_{\text{FlowMM}} = \text{AtomTypes} \times \text{FracCoords} \times \text{LatticeParams}.$$

To adapt it for polymer structure generation, we modify this to

$$\mathcal{M}_{\text{poly}} = \text{FracCoords} \times \text{zHeight}$$

thereby reducing the dimensionality of the manifold for our problem to a manifold of size $((N \times 3) + 1)$ for a chain with N atoms.

An additional layernorm was also added after the global mean and summation block to maintain stability during training.

This polymer-enabled FlowMM was trained on PolyStructChains with an effective batch size of 128 for up to 3000 epochs. Although FlowMM performs well on small crystal unit cells compared to other models such as DiffCSP and CDVAE, we find that it struggles when

predicting polymer structures, none of the samples met our generation filtration criteria. .

Figure 5 shows example structural comparisons between our polymer-enabled FlowMM and PolyGen.

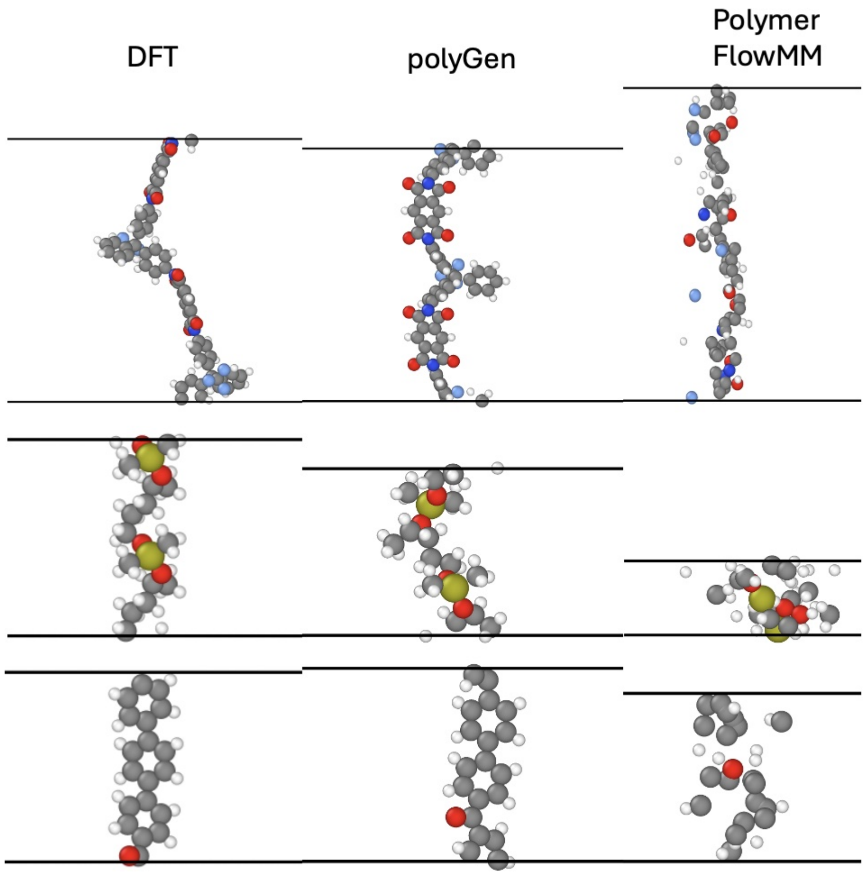

Figure 5: Structural comparisons: polymer-enabled FlowMM vs. PolyGen.

E) Polymer Substructure Presence

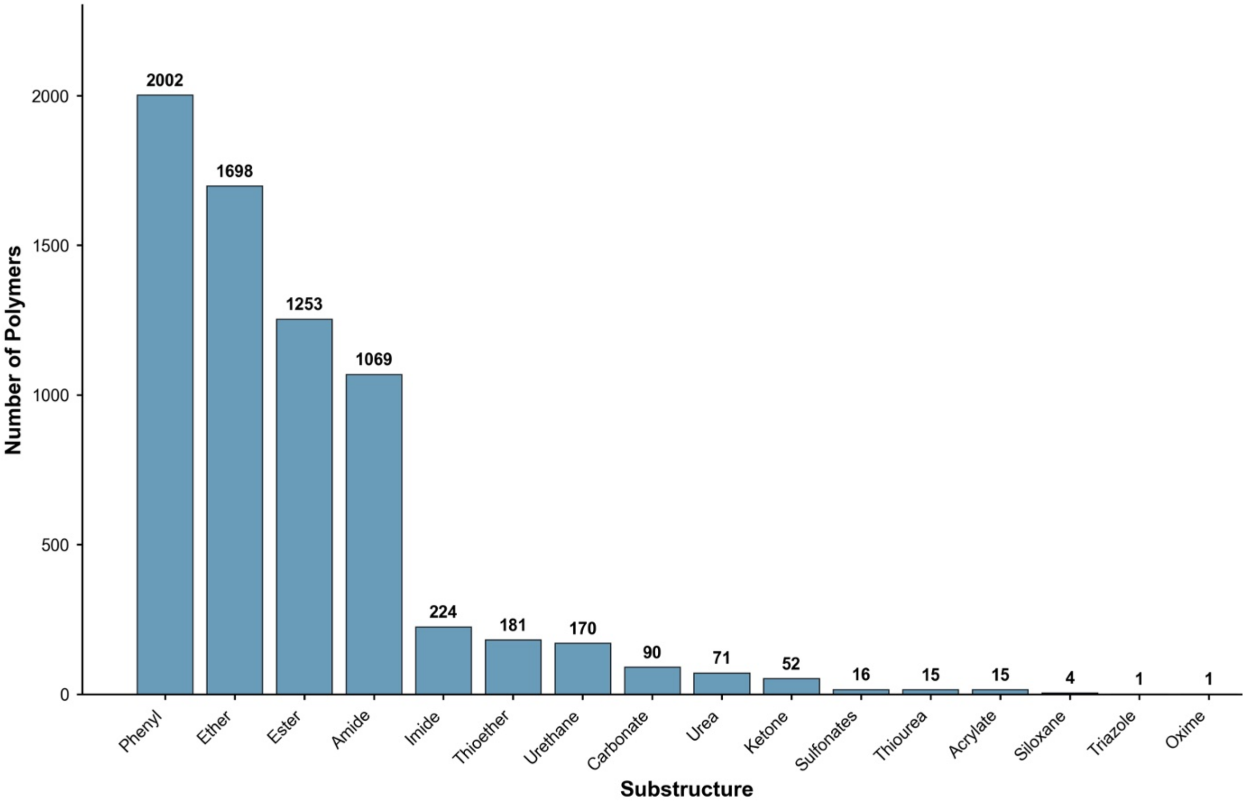

Figure 6: Chart depicting the number of polymers that contain common substructures. Each polymer can contain multiple substructures.

References

(1) Ertl, P.; Schuffenhauer, A. Estimation of synthetic accessibility score of drug-like molecules based on molecular complexity and fragment contributions. *Journal of cheminformatics* **2009**, *1*, 1–11.

(2) Hassan, M.; Shenoy, N.; Lee, J.; Stärk, H.; Thaler, S.; Beaini, D. ET-Flow: Equivariant Flow-Matching for Molecular Conformer Generation. Advances in Neural Information Processing Systems. 2024; pp 128798–128824.

(3) Karras, T.; Aittala, M.; Aila, T.; Laine, S. Elucidating the design space of diffusion-based generative models. *Advances in neural information processing systems* **2022**, *35*, 26565–26577.

(4) Joshi, C. K.; Fu, X.; Liao, Y.-L.; Gharakhanyan, V.; Miller, B. K.; Sriram, A.; Ulissi, Z. W.

All-atom Diffusion Transformers: Unified generative modelling of molecules and materials. Forty-second International Conference on Machine Learning. 2025.

(5) Miller, B. K.; Chen, R. T.; Sriram, A.; Wood, B. M. Flowmm: Generating materials with riemannian flow matching. Forty-first International Conference on Machine Learning. 2024.

(6) Jiao, R.; Huang, W.; Lin, P.; Han, J.; Chen, P.; Lu, Y.; Liu, Y. Crystal structure prediction by joint equivariant diffusion. *Advances in Neural Information Processing Systems* **2023**, *36*, 17464–17497.
